# A Latent Class Analysis of Stigmatizing Attitudes and Knowledge of HIV Risk among Youth in South Africa

**DOI:** 10.1371/journal.pone.0089915

**Published:** 2014-02-27

**Authors:** Lauren Brinkley-Rubinstein, Krista Craven

**Affiliations:** Department of Human and Organizational Development, Vanderbilt University, Nashville, Tennessee, United States of America; Federal University of Rio de Janeiro, Brazil

## Abstract

**Background:**

The current study aims to investigate how the ability to accurately gauge risk factors associated with contracting HIV while taking into consideration various individual and community level socio-demographic characteristics (e.g., race and poverty) predicts the nature of stigmatizing attitudes toward persons with HIV.

**Methods:**

Data from a sample of 1,347 Cape Town area youth who participated in the Cape Area Panel Study (CAPS) Wave 2a were used. Latent Class Analysis was conducted to ascertain whether response patterns regarding knowledge of HIV contraction suggest the presence of subgroups within the sample.

**Results:**

Findings indicate that there are four latent classes representing unique response pattern profiles regarding knowledge of HIV contraction. Additionally, our results suggest that those in South Africa who are classified as “white,” live in more affluent communities, and have more phobic perceptions of HIV risk are also more likely to have the most stigmatizing attitudes toward those who are HIV positive.

**Conclusion:**

Implications of these findings include extending HIV knowledge, education, and awareness programs to those who are not traditionally targeted in an attempt to increase levels of knowledge about HIV and, consequently, decrease stigma.

## Introduction

HIV-related stigma and resultant fears of discrimination are associated with decision making around HIV testing, screening, and seeking treatment [1]–[3]. In South Africa, which has one of the highest HIV rates in the world, stigma is pervasive among individuals and permeates cultural norms and institutions [2]. Previous research has found that knowledge related to the risk of contracting HIV, an inaccurate appraisal of one’s own HIV risk, or not knowing someone who has contracted HIV may contribute to stigmatizing attitudes [4]. For example, a 2005 study conducted among 1,746 persons aged 15 and older in the Western Cape, South Africa, found that 30% of respondents were unwilling or unsure whether they would buy food from a food seller with HIV, and 11% were unwilling or unsure whether they would care for a family member with AIDS, indicating that there may be a wide range of beliefs about the risk of contracting HIV [5].

In contrast, the more one knows about HIV, the less likely one is to have stigmatizing attitudes toward those with HIV. The contact hypothesis posits that direct interaction between individuals will lessen discrimination or stigma [6]. Research confirms this, as studies have shown that knowing someone who is HIV positive leads to a decreased probability of having stigmatizing attitudes [6]–[7]. However, Maughan-Brown (2010) found that persons with HIV in South Africa often wait until they are very sick to seek care. This may result in more negative associations because by the time these individuals are known to be HIV positive, they are likely to be very sick because of delayed care [8].

Other variables related to both interpersonal and social contexts, such as race, sex, and socioeconomic indicators, can also affect an individual’s perception of risk and one’s attitudes toward HIV positive individuals. For example, among South African men who have sex with men, perception of not being at risk for HIV infection was negatively correlated with being classified (in South Africa) as “black” or “coloured” (“coloured” is an apartheid-era term that is still used today to refer those individuals who are mixed race), knowing someone with HIV, being sexually active, and having a history of sexually transmitted infections [9]. However, there is little research involving South African populations that looks at the stratification of knowledge and stigma by demographic characteristics.

Given the distinct historical and political context of South Africa, whereby society was rigidly stratified by race and class, the current study aims to predict the presence of stigmatizing attitudes toward HIV positive populations, taking into account ability to accurately gauge risk and various individual and community level socio-demographic characteristics. Latent class analysis [LCA] is used to explain relationships among observed variables that measure knowledge of the risk of HIV contraction among homogenous classes of individuals [10]. The LCA model with covariates is then used to predict the presence of HIV-related stigma for each subgroup.

## Methods

Secondary data were used from the Cape Town Area Panel Study (CAPS), a random sample of 4,752 youth aged 14–22 from the Cape Town region of South Africa. CAPS is a longitudinal study designed and administered by a research team comprising faculty members from the University of Cape Town and the University of Michigan. Four waves of data were collected between 2002 and 2006. The current study used cross-sectional data from Wave 2a (n = 1,347), as it entailed the most comprehensive set of questions regarding knowledge of HIV/AIDS [11]. Although Wave 2a is significantly smaller in size than the overall CAPS sample, the original demographic sampling criteria of race and sex were similar between Wave 2a and the other waves (Table 1) [11].

**Table 1:** Demographic composition of entire sample and by age group.

Category	Subcategory	Overall N (%)	Age 16–18	Age 19–21	Age 22–25
Race	Black	779 (57.8%)	249	269	261
	Coloured	450 (33.4%)	159	170	121
	White	118 (8.8%)	40	44	34
Sex	Female	729 (54.1%)	240	259	230
	Male	618 (45.9%)	208	224	186
Education level	No schooling – grade 7	288 (21.4%)	200	60	28
	Grade 8–10	678 (50.3%)	247	281	150
	Grade 11–12	342 (25.4%)	1	137	204
	Post-secondary	39 (2.9%)	0	5	34
Neighborhood povertyconcentration	0–10%	175 (13.0%)	58	66	51
	11–25%	402 (29.8%)	136	154	112
	26–50%	513 (38.1%)	174	177	162
	51–75%	257 (19.1%)	80	86	91
Personally know someone withHIV/AIDS	Yes	578 (42.9%)	168	226	184
	No	769 (57.1%)	280	257	232

### Measures

#### Indicators of knowledge of HIV/AIDS risk

Knowledge of HIV/AIDS risk items includes questions that state whether an individual can get HIV by: sharing food and water, using public toilets, or engaging in protected and unprotected sexual activity (see Table 2). These items were measured using a 4-point response scale: “yes,” “no,” “maybe,” and “don’t know.” While these survey items are ordinal in nature, the majority of latent class analyses thus far have focused primarily on the use of binary and continuous variables for the composition of latent categorical variables [10]. Dichotomizing ordinal variables is thus a common practice employed when using latent class analysis to ensure interpretability of findings. Therefore, the items for this study were recoded into binary variables. Those who responded “yes,” “maybe,” or “don’t know” were coded into one category of “agree or unsure” (1), and those who responded “no” were recoded into the other category of “disagree” (0). The authors chose this variable coding to assess the difference between persons who are certain that specific risk factors do *not* increase risk of HIV versus others. (For example, this coding denotes the difference between those who are “certain” that you cannot contract HIV by using a public toilet and those who either think that you can or are unsure.).

**Table 2:** Indicators of HIV/AIDS knowledge.

Item	% total sample in disagreement with statement
“Someone with one sexual partner who does not use condoms is more likely to get HIV than someonewith many partners who always uses condoms”	28.4%
“Do you think you can get HIV by using a public toilet?”	88.3%
“Do you think you can get HIV by sharing a bath?”	91.8%
“Do you think you can get HIV by sharing a bottle of water”	89.2%
“Do you think you can get HIV by kissing on the lips?”	78.7%
“Do you think you can get HIV by deep kissing? (Putting your tongue in their mouth?)”	47.9%
“Do you think you can get HIV by touching someone’s genitals (penis or vagina) with your hand?”	56.1%
“Do you think you can get HIV by having sexual intercourse with a condom?”	54.9%
“Do you think you can get HIV by shaking hands?”	97.0%
“Do you think you can get HIV by having oral sex?”	18.7%
“In your opinion, do condoms make sex safe?”	15.7%

#### Covariates

Race, sex and knowing someone with HIV/AIDS were coded as binary variables. Blacks have experienced the highest rates of HIV and AIDS in South Africa, and thus were coded as the reference group of interest (5). Blacks were compared separately to those who are classified in South Africa as white and those who are classified as coloured. Females and those who did not have a personal connection to someone with HIV/AIDS were also coded as reference groups. Three other covariates of interest include the percent concentration of neighborhood poverty, education level, and birth year (age), each of which are continuous variables.

The concentration of poverty variable was standardized and birth year was recoded from 1 (1979) to 9 (1989). Education level was coded from 0 (no education) to 23 (undergraduate degree), with each increment of 1 representing a proportionally higher level of education.

#### Outcome variables

Outcomes of interest included the 21 CAPS items that measure HIV/AIDS stigma. These include questions such as: “Do you think it should be illegal for people with HIV/AIDS to put others at risk of infection through unprotected sex?” or “Would you be willing to look after a close family member with AIDS?” These variables were recoded from 4-point scales (“definitely or probably yes,” “definitely or probably no”) into binary outcomes of “yes” and “no”, employing the same logic as previously discussed for the interpretability of LCS analyses. In the analysis, the authors focused on propensity to agree with each item statement.

### Data Analysis

Latent class analysis (LCA) was used to uncover underlying patterns among observed variables that measure knowledge of HIV/AIDS risk. A latent categorical variable, *C*, represents the underlying relationship between these observed variables, and different levels of this latent categorical variable, *k*, are referred to as “classes” [12]. Each of these classes consists of different probabilities of endorsement for each of the 11 observed items measuring knowledge of HIV/AIDS risk. This endorsement probability is estimated by a threshold value that is generated for each item within each class. If an individual exceeds the threshold value, then the individual is likely to be in agreement with the item statement [10].

The first stage of analysis involved the examination of unconditional latent class models with different numbers of classes. The best fitting model is determined by comparing Akaike Information Criteria (AIC) to Bayesian Information Criteria (BIC) values for models with different numbers of classes. The model with the lowest BIC value is determined to best approximate the class structure of the population. Entropy statistics were also examined to determine the classification accuracy of individuals into population subgroups [10].

In the second stage of analysis, the authors examined how covariates predict class membership. To determine if the covariates of race, sex, age, education level, neighborhood poverty concentration, and knowing someone with HIV/AIDS explain class membership, a likelihood ratio test (LRT) was conducted to compare this conditional model with covariates to the unconditional LCA model.

Finally, during the third stage of analysis, the authors examined 21 outcome variables that measure HIV/AIDS stigma [11]. These outcome variables were examined in relation to the LCA model with covariates. The Wald overall test was used to determine whether the item coefficients for these outcome variables in the model are significantly different from each other across classes. Mplus 6® software (Muthén & Muthén, Los Angeles, California, USA) was used to conduct each of the aforementioned latent class analyses, and SPSS software (International Business Machines, Armonk, New York, USA) was used for cleaning the data and running descriptive analyses.

**Figure 1:**
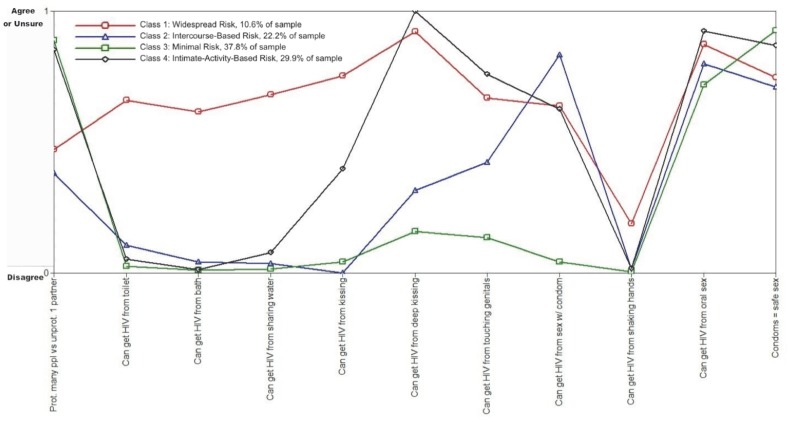
Four class unconditional latent class analysis of HIV/AIDS knowledge. Latent class analysis was used to uncover underlying patterns among observed variables that measure knowledge of HIV/AIDS risk. A latent categorical variable, *C*, represents the underlying relationship between these observed variables, and different levels of this latent categorical variable, *k*, are referred to as “classes.” Each of the four latent classes in the figure above consists of different probabilities of endorsement for each of the 11 observed items measuring knowledge of HIV/AIDS risk. Classes were labeled based on the response patterns for each latent grouping as follows: perceived widespread risk (class 1), intercourse-based risk (class 2), perceived minimal risk (class 3), and intimate activity-based risk (class 4).

Seven unconditional models ranging from two to eight classes were compared to one another using AIC and BIC to determine the appropriate class structure (Table 3). The AIC value was lowest for the 8-class model and the BIC value was lowest for the 4-class model. However, BIC typically is considered a better measure of model fit because it penalizes for model complexity more than AIC [13]. Moreover, the 4-class model provides a more interpretable and distinct set of classes than the 8-class model. Thus, we retained the 4-class model based on parsimony and substantive interpretation (Figure 1).

**Table 3:** Fit statistics.

Number of classes	AIC	BIC
2	12712.3	12832.1
3	12397.9	12580.1
4	12228.4	**12473.0**
5	12209.0	12516.1
6	12185.5	12555.1
7	12175.2	12607.3
8	**12173.2**	12667.7

## Results

Our analyses reveal that there are varying levels of knowledge regarding the risk of contracting HIV (Table 2). The majority of respondents do not think there is a risk of contracting HIV from non-sexual social contact (e.g., using a public toilet). Many respondents also do not think there is a risk of contracting HIV through non-penetrative or protected penetrative sexual contact (e.g., touching genitals, use of condoms).

The overall entropy statistic, which signifies latent class separation, was relatively high (0.75). However, classification certainty for modal class assignment was very high for class 1 (0.91) and class 3 (0.91), and moderately high for class 2 (0.82) and class 4 (0.82). These entropy statistics suggest that the response patterns of each individual in the sample could, for the most part, be clearly classified into each latent subgroup.

### Class 1: Perceived Widespread Risk

Individuals in Class 1 (10.2% of the sample population) are more likely to agree or be unsure that the risk of contracting HIV is high for activities such as sharing water or a bath, using a public toilet, kissing, touching genitals, oral sex, and protected penetrative sex, with the exception of shaking hands (Table 4). Members of Class 1 are more likely to agree or be unsure that condom use is important in practicing safe sex, and there is a 52.8% probability that individuals in Class 1 believe that protected sex with multiple partners puts one at greater risk for contracting HIV than unprotected sex with one partner. This class tends to identify the risk of HIV contraction as generally high, no matter what the activity.

**Table 4:** Probability of disagreement with statement by latent class.

Item	Class 1: Perceived widespread risk	Class 2: Intercourse-based risk	Class 3:Perceived minimal risk	Class 4:Intimate activity-based risk
“Someone with one sexual partner who does not usecondoms is more likely to get HIV than someonewith many partners who always uses condoms”	52.8%*	62.0%*	11.2%*	14.9%*
“Do you think you can get HIV by using a public toilet?”	34.0%*	89.5%*	97.6%*	94.8%*
“Do you think you can get HIV by sharing a bath?”	38.5%*	95.8%*	99.0%	98.7%
“Do you think you can get HIV by sharing a bottleof water”	31.9%*	96.4%*	98.6%*	92.1%*
“Do you think you can get HIV by kissing on the lips?”	24.7%*	100.0%	95.7%*	60.3%*
“Do you think you can get HIV by deep kissing?(Putting your tongue in their mouth?)”	7.9%*	68.6%*	84.2%*	0%
“Do you think you can get HIV by touching someone’sgenitals (penis or vagina) with your hand?”	33.1%*	57.8%*	86.5%*	24%*
“Do you think you can get HIV by having sexualintercourse with a condom?”	36.1%*	16.8%*	95.9%	37.5%*
“Do you think you can get HIV by shaking hands?”	81.1%*	98.4%	99.6%	98.3%*
“Do you think you can get HIV by having oral sex?”	12.8%*	20.0%*	28.2%*	7.7%*
“In your opinion, do condoms make sex safe?”	25.3%*	29.0%*	7.4%*	13.0%*

*p<0.05.

### Classes 2 and 4: Intercourse-Based Risk and Intimate Activity-Based Risk

Class 2 (*intercourse-based risk)* (22.2% of the sample population) and Class 4 (*intimate activity-based risk)* (29.9% of the sample population) are similar in that they are both more likely to think that one cannot contract HIV from sharing water or a bath, using a public toilet, or shaking hands (Table 4). Moreover, individuals in both these classes are unlikely to disagree that one can contract HIV via oral sex and that condom use is important in practicing safe sex. However, individuals in Class 2 are much more likely than their counterparts in Class 4 to think that protected sex with multiple partners is more risky than unprotected sex with one partner. Members of Class 2 are less likely to disagree that one can contract HIV from protected sex with a condom, but are more likely to disagree that one cannot contract HIV by touching genitals or kissing. In comparison, while members of Class 4 are also less likely to think that one cannot contract HIV from protected sex with a condom, they are less likely than members of Class 2 to think that touching genitals and kissing will not put one at risk of contracting HIV. In other words, we find that members of Class 4 are more likely to associate the risk of HIV contraction with more forms of intimate activity than are members of Class 2.

### Class 3: Perceived Minimal Risk

Members of Class 3 (37.8% of the sample population) are highly likely to disagree that one is at risk of contracting HIV from sharing water or a bath, using a public toilet, shaking hands, kissing, touching genitals, and protected penetrative sex (Table 4). Members of this class are likely to agree or be unsure that condom use is important in practicing safe sex and that there is a high risk of contracting HIV from oral sex. Additionally, individuals in Class 3 are more likely to believe that unprotected sex with one partner is more risky than protected sex with multiple partners.

### The Role of Demographic Characteristics in Predicting Class Membership

The likelihood ratio test rejected the null hypothesis that the unrestricted (conditional) and restricted (unconditional) models were equivalent, and, thus, the conditional model was retained. In other words, the conditional model with the six covariates is useful in explaining class membership.

A multinomial logistic regression was conducted to predict the probability of class *k* (widespread risk class, intercourse-based risk class, or intimate activity-based risk class) membership versus the reference class (minimal risk class) membership with the six demographic covariates (Table 5). In comparison to the perceived minimal risk class (Class 3), the perceived widespread risk class (Class 1) is approximately 17 times more likely to be coloured than black and almost 330 times more likely to be white than black (although this odds ratio is large, it was not statistically significant, likely due to lower power because of reduced *N* in certain classes). The perceived widespread risk class is also more likely to live in a neighborhood with a greater concentration of poverty, to know someone who is HIV positive, and to be male. The intercourse-based risk class is also much more likely than the perceived minimal risk class to be coloured or white than black and more likely to know an HIV positive person, but is less likely to be male, older, or live in a neighborhood with a greater concentration of poverty. Members of the intimate activity-based risk class are more likely than the perceived minimal risk class to be white or coloured than black, but these odds ratios for race are much lower than the perceived widespread risk class and the intercourse-based risk. The intimate activity-based risk class is also more likely than the perceived minimal risk class to live in a neighborhood with a greater concentration of poverty, to know someone who is HIV positive, and to be male.

**Table 5:** Odds ratios for membership in each class as compared to Class 3 (Perceived minimal risk class).

Covariate	Perceived widespreadrisk class	Intercourse-basedrisk class	Intimate activity-based risk class
Coloured vs. Black	17.06**	34.74**	1.35
White vs. Black	329.64**	512.35†	21.24†
Male vs. female	1.12	0.72	1.10
Younger vs. older	1.00	1.10	0.98
Higher level of education vs. lower	0.97	1.00	1.02
Personally know someone with HIV/AIDS	1.19	1.59*	1.23
Higher concentration of neighborhood poverty vs.lower concentration	1.44*	0.51**	1.66**

**p<0.05.

*p<0.10.

† Although these odds ratios are large, they were not statistically significant likely due to lower power because of reduced *N* in certain classes.

### Do Latent Classes Predict HIV- and AIDS-Related Stigma?

Results indicate the probability of item endorsement for each of the variables within each of the four classes (Table 6). The multivariate Wald chi-square test was significant for 20 of the 21 outcomes, meaning that, with a few exceptions, members of the perceived widespread risk class tend to have the highest propensity to stigmatize individuals with HIV/AIDS.

**Table 6:** Probability of item agreement for stigma outcomes by class†.

Item	Perceived widespreadrisk class	Intercourse-based risk class	Perceived minimal risk class	Intimate activity-based risk	*X^2^*
1. “Do you think the government should provide free health care forpeople with AIDS?”	96.9%	97.9%	99.3%	98.3%	3.2
2. “Should unemployed youth who are infected with HIVget government job training?”	81.3%	92.9%	94.5%	95.8%	25.1*
3. “Should someone with AIDS who is too sick to work get a welfaregrant from the government?”	89.6%	93.0%	97.9%	95.7%	14.8*
4. “Should a woman who got AIDS from sleeping around with manymen get a welfare grant from the government?”	40.6%	56.3%	75.2%	56.9%	58.2*
5. “Would you be willing to look after a close family member with AIDS?”	89.0%	95.7%	96.8%	97.0%	12.9*
6. “Imagine that you find out that one of your friends is HIV infected.Would you still be friends with them?”	90.4%	99.6%	98.5%	96.5%	20.5*
7. “Would you drink from the same bottle of water as an HIVinfected friend?”	26.4%	84.2%	92.5%	80.0%	174.3*
8. “If you knew that a shopkeeper had HIV, would you buy freshvegetables from him or her?”	45.6%	87.5%	92.4%	86.4%	117.8*
9. “Do you think it should be illegal for people with HIV/AIDS to putothers at risk of infection through unprotected sex?”	71.0%	71.5%	64.1%	74.6%	9.7*
10. “Do you think people with HIV/AIDS should have to disclose theirHIV status to the person they are going to have sex with *even if they* *use a condom*?”	86.9%	84.9%	80.7%	89.5%	11.2*
11. “Imagine you meet someone you really like and he/she tells youthat he/she is HIV positive, would you still go out on a “date”with him/her?”	62.3%	80.8%	91.8%	88.6%	59.1*
12. “If you loved an HIV positive person, would you have sex with themusing a condom?”	31.8%	19.4%	83.0%	77.0%	240.5*
13. “Would you prefer to know who has HIV/AIDS in your communityso that you can be careful not to get infected by them?”	85.8%	67.2%	71.2%	56.7%	31.0*
14. “Do you worry that HIV is much easier to catch than we are told?”	72.8%	63.5%	45.8%	48.6%	37.0*
15. “Would you rather not touch someone with HIV/AIDS becauseyou are scared of infection?”	46.0%	19.5%	19.0%	13.2%	47.3*
16. “Do you think the names of people with HIV/AIDS should be made public?”	26.9%	14.8%	17.5%	16.0%	8.1*
17. “Do you think HIV/AIDS is a punishment for sleeping around?”	49.0%	40.0%	14.7%	21.3%	74.6*
18. “Do you think that a school pupil with HIV puts other pupils intheir class at risk of infection?”	53.3%	25.0%	6.4%	8.6%	119.1*
19. “Do you think a school pupil with HIV should be allowed to attend school?”	76.7%	94.3%	98.0%	95.0%	56.0*
20. “Do you think that many people who get HIV infected throughsex have only themselves to blame?”	61.3%	60.1%	26.7%	31.2%	91.9*
21. “Do you think that some people with HIV/AIDS want to infect other people with the virus?”	58.2%	49.3%	25.9%	38.2%	54.4*

*Wald omnibus chi**-**square p<0.05.

† For each Wald omnibus chi-square, *df* = 3.

The most striking across-class difference is for the outcome of the question: “If you loved an HIV positive person, would you have sex with them using a condom?” Those in the intercourse-based risk class are the least likely to endorse this item (19.4%), followed by the perceived widespread risk class (31.8%). Conversely, the intimate activity-based risk class (77.0%) and the perceived minimal risk class (83.0%) are much more likely to agree that they would have protected sex with an HIV positive person they loved.

Although responses regarding protected sex with an HIV positive individual are highly variable across classes, the majority of individuals in each class are much more likely to report that they would go on a date with an individual with HIV. Some other differences across class include questions regarding whether one would drink from the same bottle of water as an HIV-infected friend, whether one would buy fresh vegetables from a shopkeeper with HIV, and whether one would rather not touch someone with HIV/AIDS because they are afraid of becoming infected. Analysis of these three items suggest that those in the perceived widespread risk class are much less likely to agree that they would share water with, buy fruits and vegetables from, or touch a person with HIV/AIDS as compared to the other three classes. Finally, one of the largest differences across class is related to believing that a fellow student with HIV puts other students in the class at risk of infection. Members of the perceived widespread risk class are much more likely to endorse this item (53.3%) than their counterparts in the other classes.

Findings related to HIV/AIDS stigma among friends and family do not vary widely across classes, although they are significantly different as determined by the Wald chi-square test. The responses illustrate that, generally, most individuals are willing to take care of a close family member with AIDS and remain friends with someone who contracts HIV. Also, there is relatively high agreement across classes that an individual with HIV/AIDS should have to disclose his or her HIV status to the person with whom they are going to have sex, even when using a condom. Results also suggest that members of each class are more likely to agree that the government should provide individuals with HIV/AIDS with various sources of support.

The only outcome that was not significant via the Wald test is whether individuals with AIDS who are sick should receive free health care. There is a nearly equal level of high agreement across classes that individuals with AIDS should have access to free health care. Along similar lines, although significantly different across class, there is a generally high propensity to agree that unemployed youth with HIV should get job training from the government and that individuals with AIDS who are too sick to work should receive a welfare grant from the government. However, when the question regarding welfare is tweaked to ask if a woman who contracted HIV from “sleeping around with many men” should get a welfare grant from the government, the likelihood of agreement within classes is generally lower and varies across class. In this case, the perceived widespread risk class is least likely to agree that a woman who contracted HIV from having sexual intercourse with several men should get a welfare grant (40.6%).

Finally, there is a distinct response profile across classes with regard to perceptions of individual responsibility in relation to contracting and spreading HIV. In response to whether “HIV/AIDS is a punishment for sleeping around,” members of the perceived widespread risk class (49.0%) and the intercourse-based risk class (40.0%) have higher agreement with this statement than members of the other two classes. Similarly, members of the perceived widespread risk class (61.3%) and the intercourse-based risk class (60.1%) are more likely to agree with the statement: “Many people who get HIV infected through sex have only themselves to blame.”

## Discussion

To the authors’ knowledge, this is the first study that has investigated the characteristics associated with stigmatizing attitudes about HIV/AIDS in South Africa using latent class analysis. The findings reveal that there is a powerful connection between certain groups’ comprehension of risk factors related to HIV and the presence of stigmatizing attitudes. Through latent class analysis, the authors identified a series of profiles of knowledge of HIV risk and then examined demographic factors that made youth more or less likely to belong to each class and more or less likely to have stigmatizing attitudes toward persons with HIV infection. The results suggest that those who are not black and who are more affluent are more likely to have phobic and inaccurate perceptions of how one contracts HIV and are also more likely to have stigmatizing attitudes about people living with HIV. The results may suggest that those in the perceived widespread risk class and the intercourse-based risk class are more inclined to view HIV contraction and spread as a matter of individual responsibility and fault than those in the intimate activity-based risk class and the perceived minimal risk class.

Currently, HIV prevention, intervention, and educational outreach efforts are primarily directed at indigenous African populations. While this is necessary and warranted given the disproportionate rate of HIV among this population, it may mean that other groups thought to be at “lower risk” have misconceptions about the risk factors that lead to HIV contraction and consequently develop stigmatizing attitudes toward persons with HIV. Given the results of the current study, efforts to reduce or eradicate stigma must be aimed at all members of society because widespread stigma can be an obstacle to HIV/AIDS testing and treatment [14]–[16]. Stigma interventions should enable citizens to understand the social context that heightens individual risk for contracting infectious diseases and promote efforts to unpack stereotypes about HIV and its connection to race. Moreover, rather than considering interventions that address only HIV, holistic interventions that seek to eliminate stigma through broader processes of racial integration and community-building may be important to consider because of the apparent social barriers related to discussing HIV across races.

Our findings are made more complex by historical legacies of racism and the sociopolitical context of South Africa. Although apartheid ended over 25 years ago, remnants of its devastating impact remain. Black, white, and coloured communities in South Africa are still largely segregated. As the current study illustrates, white youth, and to a lesser extent, coloured youth, are less likely than black youth to have an accurate understanding of the risk of contracting HIV and are more likely to have stigmatizing attitudes toward those with HIV/AIDS. Because the prevalence of HIV is highest among the black population, being HIV positive may be conflated with being black or viewed as an issue affecting *only* blacks. If so, this may contribute to tension among racial groups and could act as a hindrance to building relationships across race. While this cannot be fully answered without further empirical research, we suggest that HIV stigma is an important factor to explore in attempting to understand the various factors that might contribute to the continued social and physical segregation of different racial communities in South Africa.

The existing HIV/AIDS literature points to three primary anti-stigma strategies [17]. The first is the dissemination of information that is designed to reduce ignorance about people with HIV. The second highlights policy-level changes that make discriminatory acts illegal (e.g., anti-discrimination legislation). Finally, step three involves the participation of local community members in anti-stigma efforts [17]. Based on our findings in the South African context, we contend that there is a need for HIV education intervention to recognize and address the sociopolitical environment and the legacies of racism that may still linger from the era of apartheid, in combination with the three previously identified anti-stigma strategies. Without a more holistic approach to HIV reduction, we posit that HIV stigma may be one of many factors that hinder full racial reconciliation. An intervention, then, that addresses environmental and historical factors in combination with more general stigma strategies that have been demonstrated to be efficacious could take the shape of a reconciliation program that engages white, coloured, and black communities simultaneously. The program need not be designed as a “health intervention,” but might instead address factors that contribute to continued racial tension and segregation. This may act as a first step to reducing HIV stigma, eventually working up to explicitly addressing HIV stigma as a barrier to racial reconciliation.

### Strengths, Limitations, and Future Research

Through use of LCA, this study elucidates the nature of response patterns of knowledge regarding HIV contraction among youth and demonstrates how these latent groups may predict the presence of HIV-related stigma within segments of the population. However, these findings are not without limitations. The CAPS dataset is now somewhat dated and includes a pre-determined set of variables. As a result, we conceptualized the current study based on existing data rather than pre-selecting variables of interest. Furthermore, the response categories relevant to HIV risk perception were coded in such a way that we were unable to create simple coding categories of “yes” and “no,” but rather, were forced to create categories that were inclusive of less decisive categorical responses, such as “maybe” and “don’t know.” Future research related to this topic should explore the difference between the results that included more inclusive binary conceptualizations and more clear-cut binary categories of “yes” and “no.” Moreover, while historical legacies of racism and segregation stemming from apartheid may be related to HIV-related stigmatizing attitudes, the existing data within the CAPS dataset does not measure factors associated with racism within Wave 2. Therefore, further research that investigates this connection is needed. Contextual information that explores the meaning behind the findings would also aid in advancement of the complex implications and root causes of HIV stigma in South Africa.
